# Development and Validation of a Subject-Specific Coupled Model for Foot and Sports Shoe Complex: A Pilot Computational Study

**DOI:** 10.3390/bioengineering9100553

**Published:** 2022-10-14

**Authors:** Yang Song, Xuanzhen Cen, Yan Zhang, István Bíró, Yulei Ji, Yaodong Gu

**Affiliations:** 1 Faculty of Sports Science, Ningbo University, Ningbo 315211, China; 2 Doctoral School on Safety and Security Sciences, Óbuda University, 1034 Budapest, Hungary; 3 Faculty of Engineering, University of Szeged, 6724 Szeged, Hungary; 4 Research Academy of Medicine Combining Sports, Hwa Mei Hospital, University of Chinese Academy of Sciences, Ningbo 315010, China

**Keywords:** finite element modeling, foot, sports shoe, biomechanics, contact interaction, balanced standing, model validation

## Abstract

Nowadays, footwear serves an essential role in improving athletic performance and decreasing the risk of unexpected injuries in sports games. Finite element (FE) modeling is a powerful tool to reveal the biomechanical interactions between foot and footwear, and establishing a coupled foot-shoe model is the prerequisite. The purpose of this pilot study was to develop and validate a 3D FE coupled model of the foot and sports shoe complex during balanced standing. All major foot and shoe structures were constructed based on the participant’s medical CT images, and 3D gait analysis was conducted to define the loading and boundary conditions. Sensitivity analysis was applied to determine the optimum material property for shoe sole. Both the plantar and shoe sole areas were further divided into four regions for model validation, and the Bland–Altman method was used for consistency analysis between methods. The simulated peak plantar and sole pressure distribution showed good consistency with experimental pressure data, and the prediction errors were all less than 10% during balanced standing with only two exceptions (medial and lateral forefoot regions). Meanwhile, the Bland–Altman analysis demonstrated a good agreement between the two approaches. The sensitivity analysis suggested that shoe sole with Young’s modulus of 2.739 MPa presented the greatest consistency with the measured data in our scenario. The established model could be used for investing the complex biomechanical interactions between the foot and sports shoe and optimizing footwear design, after it has been fully validated in the subsequent works under different conditions.

## 1. Introduction

As the direct protective equipment for the foot, the main role of footwear is to protect it from hard and rough terrain or any external intrusion during daily locomotion [1]. Meanwhile, the rapid advance of the footwear industry in the past few decades has given the shoe more functions. To be specific, sports shoes with excellent cushioning, energy return, and arch support features could serve to improve athletic performance and decrease the risk of unexpected injuries in various sports games [2]. For instance, Nike Vaporfly 4% was demonstrated to improve running economy up to 6% because of the thicker midsole and curved carbon fiber plate which may contribute to a “teeter-totter effect” and significantly strengthen the energy return function [3,4]. Nevertheless, it was also clarified that exercising for a long-time while wearing inappropriate sports shoes would increase the odds of foot injuries. Plantar fasciitis, foot fractures, and heel pain were all found to be associated with abnormal plantar pressure concentration and overload during exercise, while the structure and material property of footwear play crucial roles in this process [5,6,7,8]. Thus, developing a comprehensive biomechanical approach to investigate the effects of shoe characteristics on plantar variables and the foot-shoe-ground interactions could not only help to promote the optimization of footwear product design but also prevent foot injuries.

Roughly speaking, studies on sports shoes using biomechanical and sports medicine methods have been boosted since the late 1970s [9]. Various experimental equipment and techniques such as motion capture system, force plate, and insole pressure measurement system were developed for further quantified investigations [10,11,12]. While direct experiments increase the biomechanics knowledge of foot–footwear interactions, changes of the internal stress and strain of the foot structures (e.g., bony, ligament, and soft tissue) during the interaction are unmeasurable. In this scenario, computational simulation methods such as finite element (FE) analysis have been increasingly applied for biomechanical analysis because of their capability of revealing the internal states within bony structures under different loading conditions [13,14,15]. A large number of 2D and 3D skeleton models have been developed over the past few decades [16,17,18,19]. Nevertheless, it is surprising to find out that most of the previous simulations were made for the interaction between foot and ground without considering the effects of shoe assembly, which is probably due to the structural complexity and multiple coupling conditions. It is worth noting that the foot shape will deform during locomotion, and thus, the shoe features and foot-shoe interaction will certainly influence the stress and strain characteristics of the foot. To improve accuracy, some researchers did incorporate the shoe components during simulation, while most of their models simplify its geometric structure and boundary conditions, which on the contrary, may make it less realistic and limit the further utility [20,21,22]. For instance, Li et al. (2018) [20] built a foot-barefoot running shoe FE model for landing impact analysis, but the actual structure of the barefoot running shoe and the internal space between the foot and shoe were not further considered in their study. In addition, most of the above models were validated using experimental peak plantar pressure data, while neglecting shoe sole pressure. Moreover, few studies have made further validation by dividing the corresponding areas into several more specific sites.

Therefore, the main purposes of this study were to first construct a subject-specific foot-sports shoe FE model under frictional-coupled condition and then evaluate its validity through comparison with both plantar and sole pressure of several specific regions during balanced standing, which could potentially help to update the simulation approach for foot–shoe–ground interaction and set the reference for subsequent studies aiming at dynamic foot-sports shoe FE analysis of walking and running.

## 2. Materials and Methods

### 2.1. Participant Information

A habitual rearfoot strike runner (male, age: 27 years, height: 175 cm, mass: 70 kg) with five years of running experience was involved in this study. The participant reported no prior history of lower limb injuries or foot abnormalities nor any orthopedic surgeries in the past six months. He was informed of the experiment content and signed the consent form. Ethics approval for this study was granted by the Human Subject Ethics Committee of the University.

### 2.2. Model Construction

**Figure 1:**
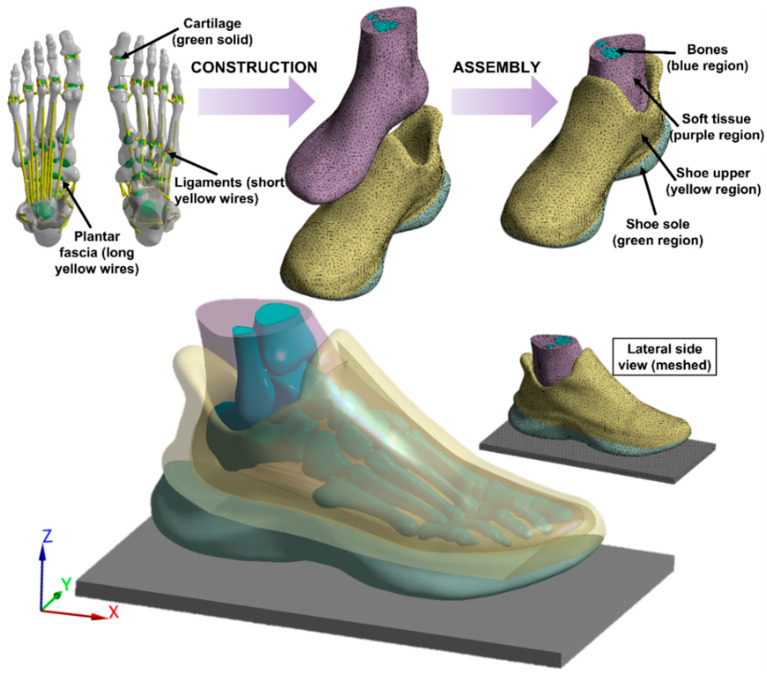
Three-dimensional finite element model of the foot and sports shoe complex. The model started from the reconstruction of each solid part of the foot and shoe, and then, the main two parts (foot and shoe) were assembled together to form the finial structure, which includes foot bone, cartilage, ligament, plantar fascia, soft tissue, shoe upper, and shoe sole.

The medical CT images (1.25 mm space interval, Optima CT540, GE Healthcare, Chicago, IL, USA) of the foot and sports shoe were collected through scanning the participant’s right leg (shod) which was fixed by an ankle-foot orthosis to the neutral position [23]. The DICOM images were segmented by MIMIC 21.0 (Materialise, Leuven, Belgium) to obtain the boundaries of bones, soft tissues, and shoe and build the 3D geometry model. The noise pixels between soft tissue and shoe cavity were manually deleted while keeping the shoe contour and thicknesses the same as their real counterpart. To reduce the computation, the second to fifth intermediate and distal phalanges were fused to one bony structure; the sock structure was considered but not separated from the soft tissue, and the shoe model was divided into two parts which stand for the shoe upper and the shoe sole, respectively. These geometries were smoothed using Geomagic Wrap 2017 (3D Systems. Rock Hill, SC, USA) and then imported into Solidworks 2020 (Dassault Systèmes, Pari, France) to form solid parts. Twenty cartilaginous structures were modelled for articulations between 20 bones considered in this model (distal parts of tibia and fibula, talus, calcaneus, cuboid, navicular, 3 cuneiforms, 5 metatarsals, and 6 phalanges) to allow the connection and relative movements. The encapsulated soft tissue was further obtained by subtracting all the bony and cartilaginous structures from the full soft tissue volume. A total of 66 ligaments and 5 plantar fasciae were created using tension-only link elements based on the anatomical locations on corresponding bones. The foot was finally assembled with the shoe to achieve the coupled foot-sports shoe model, as shown in Figure 1.

In terms of the mash process, a convergence analysis was applied to ensure both the model accuracy and the optimum requirement on computational resources [16]. Virtual topology was conducted to adapt the surface of each component. Except for the ground plate, which was meshed with hexahedrons, all other components meshed with tetrahedral solid elements. The mash size was 5.0 mm for the soft tissue, shoe upper, shoe sole, and plate; 3.5 mm for the bones; and 2.0 mm for the cartilage, and in total, there are 358,322 nodes and 208,225 elements for the whole model.

### 2.3. Material Properties

All the materials assigned in this FE analysis were idealized to be homogeneous, isotropic, and linearly elastic, and two material constants (i.e., Young’s modulus (E) and Poisson’s ratio (ν)) were used to define the elasticity. A further material property sensitivity analysis of shoe sole stiffness was conducted since this part was considered as a whole component in our simulation. Young’s modulus (E) of shoe sole was adjusted by ±10% and ±20% from the baseline value (2.490 MPa), which was obtained from previous literature [9,20,22]. The details are listed in Table 1.

### 2.4. Boundary and Loading Conditions

**Figure 2:**
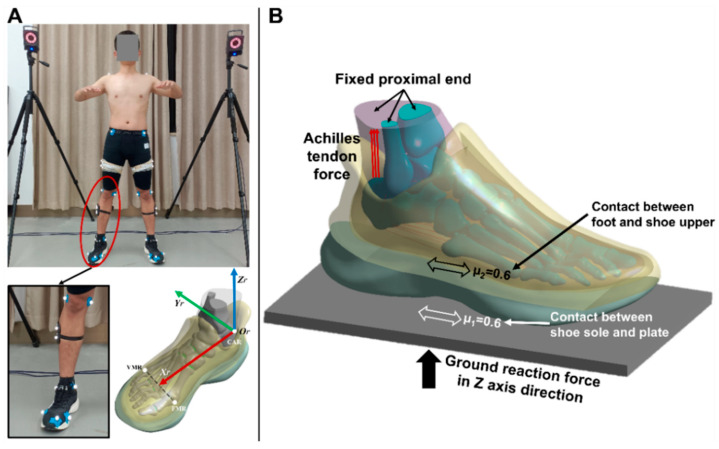
The application of boundary and loading conditions: (**A**) the heading angles of foot in sagittal and coronal plane were further calculated based on the Euler angles of foot rigid body coordinate system with respect to the global coordinate system; (**B**) the foot-plate system approach used to simulate the interaction between the foot, shoe, and ground, μ1 represents the friction coefficient between the shoe sole and plate while μ2 represents the friction coefficient between the foot and shoe upper.

Three-dimensional gait analysis was carried out on the same participant using an 8-camera Vicon motion capture system (Oxford Metrics Ltd., Oxford, UK) and AMTI force platform (Advance Mechanical Technology Inc., Watertown, NY, USA) synchronously, in which the boundary and loading conditions were determined. Specifically, a total of 48 reflective markers were attached to the corresponding bony landmarks according to the previously established protocol [24] (Figure 2A), and the balanced standing trial was conducted when the subject stood on the force plate with his right leg. The vertical ground reaction force (vGRF) was derived, and the heading angles of foot in sagittal and coronal plane were further calculated based on the Euler angles (γ = −4.73°; β = −1.15°) of foot rigid body coordinate system with respect to the global coordinate system (Figure 2A) because the plantar pressures were highly associated with the foot orientation during simulation [25].

In this study, a foot-plate system approach was used to simulate the interaction between the foot, shoe, and ground (Figure 2B). First, a three-dimensional solid plate that was allowed to move only in the vertical direction was used to model the supporting ground, and the proximal surfaces of the soft tissue, tibia, and fibula components were fixed. Moreover, two additional forces were assigned to the model. One is the vGRF, which was applied at the inferior surface of the ground plate (343.00N). The other is the Achilles tendon force, which has been estimated as half of the ground support force during standing and was applied at the superior surface of the calcaneus (171.50N). In terms of the interaction between the foot, shoe, and ground plate, both the connection types were defined as the frictional contact with a coefficient of 0.6.

### 2.5. Model Validation

To validate the coupled foot-shoe model, the plantar and sole pressure from the computational simulation were compared with the experimental data collected from the same participant using the Novel Pedar-X insole pressure measurement system and Novel Emed force plate measurement system (Novel GmbH, Munich, Germany), respectively. Both the plantar and sole areas were further divided into four specific regions, including medial forefoot (MFF), lateral forefoot (LFF), midfoot (MF), and hindfoot (HF) for foot model, medial fore-sole (MFS), lateral fore-sole (LFS), medial hind-sole (MHS), and lateral hind-sole (LHS) for the shoe model. The pressure distribution and predicted peak pressure of all eight regions were validated against the corresponding experimental pressure. Moreover, the Bland–Altman method was further performed to calculate the difference and mean of the pressure data obtained from experiment and model simulation through MedCalc 19.0.4 (MedCalc Software, Ostend, Belgium). The two approaches were considered as presenting good consistency if the difference was within the 95% limits of agreement (LOA).

## 3. Results

### 3.1. Plantar Pressure Validation

**Figure 3:**
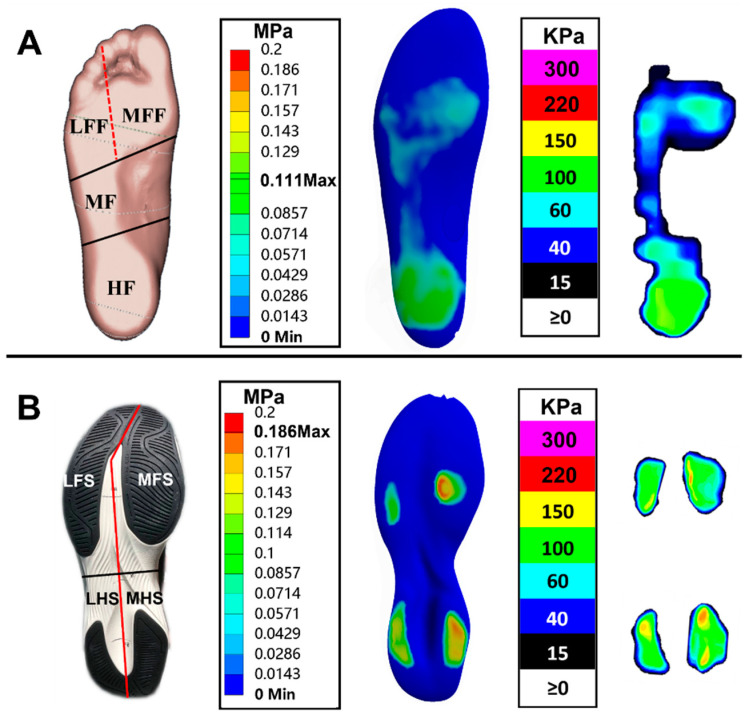
The subdivided regions and comparison between predicted pressure distribution and experimental pressure data: (**A**) plantar region and (**B**) sole region; both the plantar and sole areas were further divided into four specific regions, including medial forefoot (MFF), lateral forefoot (LFF), midfoot (MF), and hindfoot (HF) for foot model, medial fore-sole (MFS), lateral fore-sole (LFS), medial hind-sole (MHS), and lateral hind-sole (LHS) for shoe model.

Figure 3A shows the subdivided plantar regions and the comparison between predicted plantar pressure distribution and corresponding measured insole pressure data during balanced standing. The highest plantar pressure was located at the HF region and followed by the MFF, LFF, and MF regions, which presented a good consistency with the experimental pressure data. The predicted and measured plantar pressure data, the corresponding relative error, and the total average error are given in Table 2. The peak pressure relative errors were less than 10% both in MF and HF regions while they increased considerably in the MFF and LFF regions, which further led to a relatively high average error.

### 3.2. Sole Pressure Validation

Figure 3B shows the subdivided shoe sole regions and the comparison between predicted sole pressure distribution and corresponding measured plate pressure data during balanced standing. The peak pressure was mainly concentrated on the medial regions of the shoe sole (i.e., MFS and MHS regions) and followed by LFS and LHS regions, which was consistent with the measured data. The predicted and measured sole pressure values, the relative and total average error, and the sensitivity analysis results for shoe sole material properties are listed in Table 3. The peak pressure relative errors were all below 10% in the 4 shoe sole regions with the baseline material property.

In terms of the sensitivity analysis, distinct changes were exhibited in peak pressure when shoe sole material properties changed (Table 3). Specifically, the peak pressure greatly increased with the hardened shoe sole and decreased with the softened shoe sole. The shoe sole material with a 10% increased Young’s modulus (E) presented a consistent relative error with baseline when compared to experimental data, while other cases resulted in percentage changes over 10%. All the average errors were less than 10%, with only one exception (13.49% with (baseline − 20%) material property). In summary, shoe sole with a Young’s modulus of 2.739 MPa (baseline + 10%) presented the greatest consistency with the experimental pressure data.

### 3.3. Agreement Analysis

**Figure 4:**
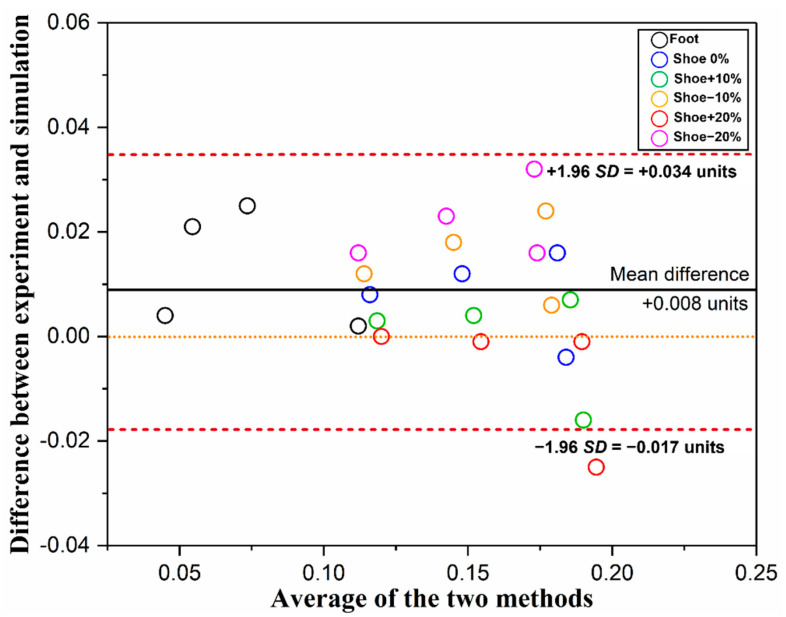
Bland–Altman plot of experimental and simulated pressure data. Different colored circles represent different regions. As shown in the figure legend, the black one represents the plantar regions, while the other five represent the sole regions with different material properties.

As it is shown in Figure 4, the Bland–Altman plot presented the mean difference and 95% LOA between the experimental and simulated pressure data. The mean difference (+0.008) is very close to 0, as indicated by the solid line. Moreover, most of the points (23/24, 96%) are scattered between ± 1.96SD (red dashed line), which indicates that the two approaches are in relatively good agreement.

## 4. Discussion

Both foot and shoe shapes would undergo deformation during locomotion, and thus incorporating footwear with the foot in the model is a prerequisite to further reveal the realistic foot biomechanical responses during the interaction. Accordingly, in this study, a 3D, subject-specific coupled FE model of the foot and ankle together with the sports shoe was introduced with boundary and loading conditions defined through gait experiments of the same participant.

The validation of a FE model is a crucial issue after it was developed since it is highly associated with model accuracy and practicality. The rearfoot pressure-based approach was commonly used in most previous works [20,21]. In the current study, both the plantar and shoe sole areas were further divided into several regions for validation. The results showed that the predicted plantar and shoe sole pressure distributions exhibited good consistency with the experimental balanced standing pressure data (Figure 3). Moreover, all the relative errors for peak pressures were lower than 10% with two exceptions. Specifically, both the simulated peak pressures in the MFF and LFF regions were lower than the volunteer measurements, which consequently increases the relative error level. This discrepancy is perhaps due to the following several reasons. First, only muscle force applied on the Achilles tendon was considered in this FE model since it plays an important role during balanced standing, while other extrinsic and intrinsic foot muscle forces were neglected. Yu et al. (2008) [26] also speculated that the lower predicted pressures found at the first and fifth metatarsal heads were associated with the above force setting. Second, a relatively larger deformation of the forefoot was detected during the simulation, which may further offset part of the pressure effect from the ground reaction force. On the contrary, the foot, footwear, and ground were all in a relatively static state during the balanced standing test. Third, the resolution differences between experimental pressure measurement and computational prediction may also have some influences on the reading. Nevertheless, the results of rearfoot pressure comparison and Bland–Altman analysis between two methods demonstrated that the current FE model is useful under the balanced standing scenario, and subsequent validation study for the coupled model will be conducted under dynamic loading conditions. Meanwhile, it is worth noting that, with the accessibility of dual-plane fluoroscopy and high-resolution MRI, the FE model could be further validated against in vivo joint motion and soft tissue deformation data recorded by the two techniques [25,27].

Sensitivity analysis was also performed in this study to determine the effects of shoe sole material property on pressure. The peak pressure greatly increased with hardened shoe sole and vice versa with softened one, and it was found that shoe sole with Young’s modulus of 2.739 MPa, which is between Young’s modulus of ethylene-vinyl acetate (EVA, 1.000 MPa) and thermoplastic polyurethane (TPU, 3.000 MPa), was the most suitable setting in this simulation [9]. In some of the previous studies, nonlinear hyperelastic material defined by the five-term Moonley–Rivlin model was used for the outsole model to simulate the rubber-like material behavior [9,20]. Although it is much closer to the actual outsole material property, some challenges of utilizing hyperelastic material for shoe soles still need to be overcome. For example, it is challenging to define the outsole using hyperelastic material if the whole shoe sole was fused into one assembly. Moreover, both the soft tissue and shoe sole were defined by linear elastic material to reduce the computational demands especially incurred by the intensive sensitivity tests. The model run time would become excessive when shifting the material to the nonlinear one and consequently reduce the efficiency. Nevertheless, recent technological advances in medical imaging demonstrated the possibility to obtain personalized nonlinear material property data, which indicates promising directions for future research [28,29].

Several previous publications have also developed the coupled foot-shoe FE model intending to observe the internal changes within bony structures under different foot motions. A foot-boot model was constructed by Qiu et al. (2011) [21] and validated through published data during balanced standing, and it was further used for the simulation of the military parachute landing. Cho et al. (2009) [9] developed a 3D coupled foot-sports shoe model to analyze the mutual interaction between foot and footwear during landing and further assess its reliability through comparison between predicted results and experimental data. Similarly, a recent paper by Li et al. (2019) [20] constructed a coupled FE model of the foot and barefoot running footwear to investigate the plantar pressure differences between the barefoot model and the coupled model during the weight-bearing moment of running. Nevertheless, all these previous shoe models were built based on the contour profile of the ankle and foot model rather than the real structure. On the other hand, some foot models were over-simplified to one bone assembly. Although some details were also simplified in our model (e.g., shoelace and insole), the most prominent feature of our model was the realization of foot–shoe interface based on the actual characteristics, which could be used to simulate various kinds of foot motion and reproduce more realistic interaction effects between foot and footwear in virtual circumstances [30,31,32]. Improvement of other sports shoe geometry designs could be made to the coupled model based on the specific purposes of future simulations. Additionally, it must be noted that, currently, the subject-specific approach is still a typical study design for FE analysis, which may further hinder the generalizability of the findings. Despite that, some studies have applied the population-based models, but this approach may not be feasible in all scenarios (e.g., more sophisticated models or models under complex boundary and loading conditions). Further studies are likely to solve this technical problem.

## 5. Conclusions

In this study, a fully coupled 3D foot-sports shoe FE model has been proposed. The FE coupled model could further serve to reveal the complex biomechanical interactions between the foot and the sports shoe and optimize footwear design after full validation both under static and dynamic scenarios.

## Figures and Tables

**Table 1:** Material properties assigned to each component in the finite element model.

Component	Element Type	Young’s Modulus E (MPa)	Poisson’s Ratio ν	Cross-Section Area (mm^2^)	Mass Density ρ (kg/m^3^)
Shoe upper	Tetrahedral solid	11.76	0.35	-	9400
Shoe sole (baseline)	Tetrahedral solid	2.49	0.35	-	2300
Bone	Tetrahedral solid	7300	0.30	-	1500
Cartilage	Tetrahedral solid	1	0.40	-	1050
Ligament	Tension-only truss	260	0.40	18.4	937
Plantar fascia	Tension-only truss	350	0.40	58.6	937
Soft tissue	Tetrahedral solid	1.15	0.49	-	937
Ground plate	Hexahedral solid	17,000	0.10	-	5000

**Table 2:** Comparison of predicted peak plantar pressures and experimental pressure insole data in 4 plantar regions during balanced standing.

Plantar Region	Peak Plantar Pressure (MPa)		
	Experiment	Simulation	Relative Error (%)
MFF	0.086	0.061	−29.07
LFF	0.065	0.044	−32.21
MF	0.047	0.043	−8.51
HF	0.113	0.111	−1.77
Average error (%)	$\overset{4}{\underset{n=1}{\sum }}\left|Pi\right|$ = 17.89		

Note: medial forefoot (MFF), lateral forefoot (LFF), midfoot (MF), and hindfoot (HF).

**Table 3:** Comparison of predicted peak sole pressures and experimental pressure plate data in 4 sole regions during balanced standing.

Sole Region	Peak Sole Pressure (MPa)										
	Experiment	Simulation (Baseline)		Simulation (Baseline + 10%)		Simulation (Baseline + 20%)		Simulation (Baseline − 10%)		Simulation (Baseline − 20%)	
	Value	Value	Relative Error %	Value	Relative Error %	Value	Relative Error %	Value	Relative Error %	Value	Relative Error %
MFS	0.182	0.186	2.20	0.198	8.79	0.207	13.74	0.176	−3.30	0.166	−8.79
LFS	0.120	0.112	−6.67	0.117	−2.50	0.120	0.00	0.108	−10.00	0.104	−13.33
MHS	0.189	0.173	−8.47	0.182	−3.70	0.190	0.53	0.165	−12.70	0.157	−16.93
LHS	0.154	0.142	−7.79	0.150	−2.60	0.155	0.65	0.136	−11.69	0.131	−14.94
Average error (%)		$\overset{4}{\underset{n=1}{\sum }}\left|Pi\right|$ = 6.28		$\overset{4}{\underset{n=1}{\sum }}\left|Pi\right|$ = 4.39		$\overset{4}{\underset{n=1}{\sum }}\left|Pi\right|$ = 3.73		$\overset{4}{\underset{n=1}{\sum }}\left|Pi\right|$ = 9.42		$\overset{4}{\underset{n=1}{\sum }}\left|Pi\right|$ = 13.49	

Note: medial fore-sole (MFS), lateral fore-sole (LFS), medial hind-sole (MHS), and lateral hind-sole (LHS).

## Data Availability

Data are available on request due to ethical restrictions.
